# The role of enteric hormone GLP-2 in the response of bone markers to a mixed meal in postmenopausal women with type 2 diabetes mellitus

**DOI:** 10.1186/s13098-015-0006-7

**Published:** 2015-02-27

**Authors:** Laura S Girão Lopes, Rubens Prado Schwartz, Bruno Ferraz-de-Souza, Maria Elizabeth Rossi da Silva, Pedro Henrique Silveira Corrêa, Márcia Nery

**Affiliations:** Unidade de Diabetes, Serviço de Endocrinologia e Metabologia, Hospital das Clínicas da Faculdade de Medicina da Universidade de São Paulo, São Paulo, Brazil; Instituto de Radiologia, Hospital das Clínicas da Faculdade de Medicina da Universidade de São Paulo, São Paulo, Brazil; Laboratório de Carboidratos e Radioimunoensaios/LIM-18, Faculdade de Medicina da Universidade de São Paulo, São Paulo, Brazil; Unidade de Doenças Osteometabólicas do Serviço de Endocrinologia e Metabologia, Hospital das Clínicas da Faculdade de Medicina da Universidade de São Paulo, São Paulo, Brazil

**Keywords:** Bone remodeling, Type 2 diabetes mellitus, Mixed meal, GLP-2, CTX, Osteocalcin

## Abstract

**Background:**

Type 2 diabetes mellitus (T2D) is a complex disease associated with several chronic complications, including bone fragility and high fracture risk due to mechanisms not yet fully understood. The influence of the gastrointestinal tract and its hormones on bone remodeling has been demonstrated in healthy individuals. Glucagon-like peptide 2 (GLP-2), an enteric hormone secreted in response to nutrient intake, has been implicated as a mediator of nutrient effects on bone remodeling. This study aimed to analyze the dynamics of bone resorption marker C-terminal telopeptide of type I collagen (CTX), bone formation marker osteocalcin, and GLP-2 in response to a mixed meal in diabetic postmenopausal women.

**Methods:**

Forty-three postmenopausal women with osteopenia or osteoporosis (20 controls – group CO – and 23 diabetic – group T2D) were subjected to a standard mixed meal tolerance test, with determination of serum CTX, plasma osteocalcin and serum GLP-2 concentrations at baseline and 30, 60, 120 and 180 minutes after the meal.

**Results:**

T2D women had higher body mass index as well as higher femoral neck and total hip bone mineral density. At baseline, luteinizing hormone, follicle-stimulating hormone, osteocalcin and CTX levels were lower in group T2D. In response to the mixed meal, CTX and osteocalcin levels decreased and GLP-2 levels increased in both groups. The expected CTX suppression in response to the mixed meal was lower in group T2D.

**Conclusions:**

Bone turnover markers were significantly reduced in T2D women at baseline. Confirming the role of nutrient intake as a stimulating factor, GLP-2 increased in response to the mixed meal in both groups. Importantly, CTX variation in response to the mixed meal was reduced in T2D women, suggesting abnormal response of bone remodeling to nutrient intake in T2D.

## Introduction

Despite high bone mineral density (BMD), studies have shown that men and women with type 2 diabetes mellitus (T2D) are at increased risk for fracture, particularly nonvertebral fractures [1-6]. Although the pathophysiology of increased bone fragility in individuals with T2D is not fully understood, several factors such as body weight, glycemic control and the presence of chronic complications might contribute [6].

Changes in the rate of bone turnover are an important determinant of bone disease, and the development of better assays has improved the ability of bone turnover markers to reflect the rate of bone remodeling. Serum C-terminal telopeptide of type 1 collagen (CTX) is the reference serum marker for bone resorption, arising from the degradation of type I collagen in resorbed bone [7]. Osteocalcin (OC) is the most abundant non-collagenous protein found in bone, being secreted by osteoblasts during osteoid synthesis and released into the circulation, providing a marker for bone formation [8].

Biochemical markers of bone resorption follow a circadian rhythm, rising at night and falling during the day, and feeding seems to induce this decrease [9-11]. Conversely, the effects of nutrient intake on bone formation is still a subject of debate, since some authors describe no variation [12] or decrease [13] in response to nutrient intake. The role of the gastrointestinal tract and its hormones, particularly glucagon-like peptide 2 (GLP-2), in bone remodeling has been recently studied in a variety of clinical settings, including healthy volunteers, postmenopausal women and individuals with short bowel syndrome [12,14]. Another relevant issue is the reciprocal influence between bone and energy metabolism, that has been described involving osteocalcin, insulin and leptin [15].

GLP-2 is a 33 amino acid peptide derived from the post translational processing of glucagon. Biologically active GLP-2_1–33_ is secreted from the enteroendocrine L cells of the ileum and colon in response to nutritional, hormonal and neural stimulation [16]. The main physiological function of GLP-2 is its trophic action on the bowel mucosa, promoting its growth and improving its absorptive function [16,17]. More recent data implicate GLP-2 as a mediator of the effects of nutrition on bone metabolism, particularly on the suppression of bone reabsorption [12,18,19]. Accordingly, short-bowel syndrome patients without a colon showed no reduction in serum concentration of CTX when compared to normal controls, suggesting that bone resorption is decreased postprandially by intestinal factors and GLP-2 is a possible candidate [19]. In postmenopausal women, exogenous GLP-2 inhibits bone resorption [20], strengthen the evidence that GLP-2 influences bone resorption. No data are available on the influence of feeding or intestinal factors on bone remodeling in the context of insulin resistance.

The aim of this study was to assess the effect of a mixed meal on serum concentrations of bone remodeling markers OC and CTX and gastrointestinal hormone GLP-2 in postmenopausal women with T2D and low BMD.

## Materials and methods

### Study design and subjects

This study comprised postmenopausal women younger than 65 years of age and with body mass index (BMI) between 20 and 35 kg/m^2^ recruited at Hospital das Clínicas da Faculdade de Medicina da Universidade de São Paulo, a tertiary center located in the state of São Paulo, Brazil. Ethical approval was obtained from the institutional research Ethics Committee of Hospital das Clínicas da Faculdade de Medicina da Universidade de São Paulo and participants were only included in the study following written informed consent. Eligible women initially underwent a dual-energy x-ray absorptiometry (DXA) scan of the hip and lumbar spine in addition to laboratory testing to assess bone and carbohydrate metabolism, including total calcium, phosphorus, parathyroid hormone (PTH), 25-hydroxyvitamin D, serum CTX, plasmatic OC, 24-hour urinary calcium, besides serum GLP-2. Only women with low BMD, as defined by current World Health Organization guidelines as T-scores < −1.0 at either site [21] were selected for the study. Menopause was defined as the absence of menstrual cycles for a minimum of four years confirmed by measurement of follicle-stimulating hormone (FSH) above 35 mUI/mL and estradiol below 20 pg/mL. Exclusion criteria were as follows: current use of drugs known to interfere with bone metabolism, current use of hormone replacement therapy, primary bone diseases with secondary osteoporosis, kidney failure. Additionally, controle women were excluded if they had impaired fasting glucose or glucose intolerance according to American Diabetes Association (ADA) criteria [22]. Included participants were allocated to two groups: group T2D, comprising women with diagnosis of T2D and group CO comprising control women. All participants were subjected to the mixed meal tolerance test, as described below. Participants using thiazides or selective serotonin reuptake inhibitors were instructed to interrupt use of these medications 48 hours prior to the mixed meal tolerance test.

### Mixed meal tolerance test

Following 12-hour overnight fasting, subjects were offered a breakfast-like meal at 8:00 am. Blood samples were drawn at baseline and 30, 60, 120, and 180 minutes after the meal. A standard meal was given, composed of 562 kcal corresponding to 51.2% carbohydrates, 33.4% lipids and 15.4% protein, in addition to 8.2 g of monounsaturated fatty acids and 5.2 g of polyunsaturated fatty acids, and had to be consumed within 15 minutes.

### Hormonal determinations

Osteocalcin (OC) was measured using an ELISA (*enzyme-linked immunoabsorbent assay*) kit (DIAsource ImmunoAssays S.A., Belgium) [23,24]; the normal reference range is 5–25 ng/mL, with an intra-assay variation of 3–5% and an inter-assay variation of 3.5–5.6%. C-terminal telopeptide (CTX) was measured using an ELISA kit (Immuno Diagnostic Systems Ltd, United Kingdom) [25]; the normal reference range for postmenopausal women is 0.142–1.351 ng/mL, with an intra-assay variation of 1.7–3% and inter-assay variation of 3–11%. GLP-2 was measured using an ELISA kit (Millipore Corporation, United States) [26,27] with a standard curve of 1–64 ng/mL, intra-assay variation of less than 10%, and inter-assay variation of less than 12%. This assay measures total levels of GLP-2, including active [1-33] and inactive [3-33] isoforms.

### Statistical analysis

Statistical analysis was performed using SPSS v15.0, setting the level of significance was at 0.05. Numerical variables are expressed as mean ± standard deviation and were compared using Student’s *t*-test. Percent values were compared using the chi-square test, and hormonal curves were analyzed through two-way (time-point and group) analysis of variance (ANOVA) with repeated measures, followed by the Tukey’s multiple comparison test when statistically significant differences were identified between the groups [28]. Pearson correlation coefficients were used to assess the relationship between CTX, OC, GLP-2, BMD and body mass index (BMI).

## Results

### Group characteristics and baseline assessment

A total of 43 postmenopausal women with low BMD (osteoporosis or osteopenia) participated in this study, comprising 23 women with T2D (group T2D) and 20 controls (group CO). General characteristics of participants are shown in Table 1. Although the two study groups were not matched, the participants’ age and the time since menopause were similar in groups T2D and CO. The groups differed in BMI, which was greater in group T2D (30 ± 3.9 vs. 27 ± 5.3 kg/m^2^, p = 0.044), and in femoral neck and total hip BMD, which were also higher in group T2D (Table 1).

**Table 1:** **Baseline characteristics of study participants**

**Variable**	**Group T2D (n = 23)**	**Group CO (n = 20)**	**p-value**
	**Mean (SD)**	**Mean (SD)**	
Age (years)	59.8 (4.2)	57.8 (3.3)	0.087
Time since menopause (years)	9.8 (4.3)	7.4 (4.5)	0.105
Diabetes duration (years)	13.7 (9.2)	—	—
BMI (kg/m^2^)	30 (3.9)	27 (5.3)	0.044
Femoral neck BMD (g/cm^2^)	0.78 (0.09)	0.69 (0.08)	0.003
Total hip BMD (g/cm^2^)	0.93 (0.09)	0.82 (0.09)	<0.001
Lumbar spine BMD (g/cm^2^)	0.87 (0.09)	0.82 (0.12)	0.17

T2D, type 2 diabetes; CO, control; SD, standard deviation; p-value, Student’s *t*-test p-value; BMI, body mass index; BMD, bone mineral density.

Approximately 70% of the participants with diabetes also had arterial hypertension, and almost half were obese. Both diseases were less prevalent in group CO (p < 0.05). In group T2D, the median duration of diabetes was 13.7 ± 9.2 years, and mean glycated hemoglobin level (HbA1c) was 7.8 ± 1.7%. Among participants with T2D, 47.8% used insulin, and 91.3% used oral hypoglycemic agents. The most prevalent chronic complication of diabetes was macrovascular disease.

Baseline laboratory testing revealed additional differences between the groups (Table 2). Mean follicle-stimulating hormone (FSH) and luteinizing hormone (LH) levels were lower in group T2D compared to CO. Groups CO and T2D were similar in regard to the following baseline bone metabolism parameters: 25-hydroxyvitamin D, total calcium, phosphorus, PTH (parathyroid hormone), and 24-hour urinary calcium and phosphate. Baseline CTX and OC concentrations were lower in participants with T2D compared to the controls, while GLP-2 concentrations were similar in both groups (Table 2).

**Table 2:** **Baseline laboratory evaluation**

**Variable**	**Reference range**	**Groups T2D (n = 23)**	**Group CO (n = 20)**	**p-value**
		**Mean (SD)**	**Mean (SD)**	
FSH (IU/L)	31 to 134	49.7 (16.5)	78.1 (13.4)	< 0.001
LH (IU/L)	15 to 64	24.2 (12.4)	36.2 (11.4)	0.012
Estradiol (pg/mL)	<25	15.7 (2.7)	17,9 (6.0)	0.15
Total calcium (mg/dL)	8.6 to 10.2	9.6 (0.6)	9.3 (0.6)	0.129
Phosphorus (mg/dL)	2.7 to 4.5	3.9 (0.5)	3.6 (0.5)	0.164
PTH (pg/mL)	16 to 87	45.3 (18.8)	51.1 (17.0)	0.237
25-OHD (ng/mL)	30 to 100	21.3 (8.5)	20.4 (5.2)	0.685
CTX (ng/mL)	0.142 to 1.351	0.49 (0.25)	0.66 (0.22)	0.04
Osteocalcin (ng/mL)	5 to 25	10.2 (5.4)	14.8 (5.3)	0.008
GLP-2 (ng/mL)	1 to 6415	4.1 (2.0)	3.8 (1.8)	0.61
24-hour urinary calcium (mg/24 h)	100 to 320	174.7 (91.6)	151.9 (63.2)	0.44

T2D, type 2 diabetes; CO, control; SD, standard deviation; p-value, Student’s t-test p-value; FSH, follicle-stimulating hormone; LH, luteinizing hormone; PTH, parathyroid hormone; CTX, C-terminal telopeptide of type I collagen; GLP-2, Glucagon-like peptide 2.

For all 43 participants, BMI had a positive correlation with hip BMD (p = 0.004; r = 0.444). In group T2D, glycated hemoglobin (HbA1c) levels had no correlation with BMD values at any assessed sites.

### Hormonal response to the mixed meal tolerance test

**Figure 1:**
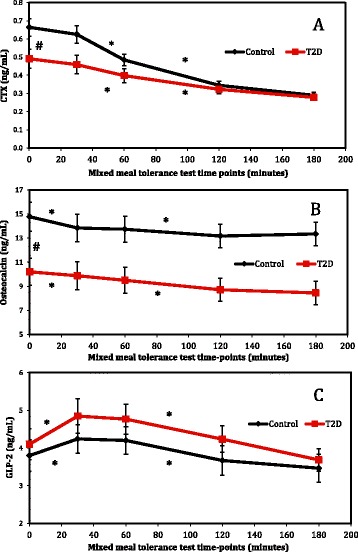
**Response of CTX, osteocalcin and GLP-2 to the mixed meal tolerance test.** Results (mean and standard error) for controls, in black, and T2D patients, in red, are shown for CTX **(A)**, osteocalcin **(B)** and GLP-2 **(C)**. *Statistically significant variation (p < 0.05 on analysis of variance – ANOVA) was observed throughout the mixed meal tolerance test #Significant difference (p < 0.05 on Tukey’s multiple comparison test) was observed between the groups at baseline.

**Figure 2:**
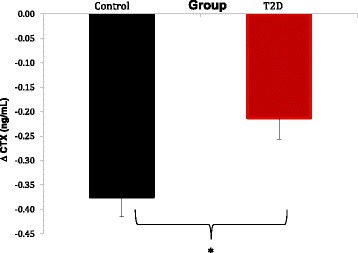
**CTX suppression in response to the mixed meal.** Variation between basal and nadir for controls (black) and T2D patients (red) are shown (mean and standard error). *Student’s *t*-test p-value = 0.006.

Serum CTX levels decreased throughout the mixed meal tolerance test in both groups (p < 0.05) (Figure 1). Intergroup comparison of test curves detected a statistically significant difference (p = 0.003), which was attributable to higher baseline values in group CO and to reduced variation of CTX in group T2D throughout the test (T2D Δ: −0.21 vs. CO Δ: −0.38 ng/mL; p < 0.050) (Figures 1 and 2).

Following the mixed meal, plasma OC levels decreased gradually in both groups (p < 0.001). OC levels were lower in diabetic participants compared to controls in all the time-points of the meal test. OC response curves were similar between groups T2D and CO (p = 0.222) (Figure 1).

GLP-2 levels varied throughout the mixed meal tolerance test in both groups (p < 0.001), increasing from baseline to time-point 30 minutes and decreasing from time-point 120 minutes onwards (p < 0.05), as shown in Figure 1. Intergroup comparison of response curves showed that the GLP-2 dynamics were similar in both groups throughout the test (p > 0.05) (Figure 1).

In all subjects, a significant negative correlation was found between OC levels at all mixed meal tolerance test time-points and BMI (p < 0.05, r value ranging from −0.339 to −0.417). Conversely, CTX levels had no correlation with BMI or BMD. Interestingly, GLP-2 levels had a positive correlation with total hip and femoral neck BMD at all the mixed meal tolerance test time-points (p < 0.05, r value ranging from 0.349 to 0.506). Inclusion of BMI as a potential confounding factor in ANOVA showed that BMI did not account for the differences in OC and CTX levels observed between groups T2D and OC.

## Discussion

This study aimed to analyze the response of bone remodeling markers and gastrointestinal hormone GLP-2 to a mixed meal in postmenopausal women with T2D in comparison to controls. We show that T2D participants had lower levels of bone turnover markers at baseline and, importantly, that the degree of suppression of bone resorption marker CTX following a mixed meal is reduced in diabetic women.

Our study design included a mixed meal tolerance test to stimulate GLP-2 production, which could potentially better reflect the effects of nutrient absorption on enteric hormones on bone remodeling markers than the oral glucose tolerance test [29] or the separate intake of various nutrients [12]. Besides its well-established role in bowel mucosa growth and function, GLP-2 has been implicated as a mediator of nutrient effects on bone resorption.

The analyzed groups were similar in regard to participants’s age and time since menopause, which are relevant factors in the study of bone metabolism parameters. Comparison of BMD between groups T2D and CO revealed greater femoral neck and total hip BMD in T2D participants, which is in agreement with the literature [3-5,30]. Nevertheless, participants with T2D had higher BMI, which could limit the interpretation of DXA assessment, since obesity is a well-know confounding factor for BMD[31]. No correlation was found between HbA1c levels and BMD among T2D participants, which is disagreement with the results of prior studies [30], but could be attributable to the small sample size.

Of note, mean baseline FSH and LH levels were higher in controls than is T2D participants, even though time since menopause was similar between groups. Ovarian function is altered in diabetic women throughout reproductive life and in menopause, as shown by an earlier decline in AMH and inhibin B levels in type 1 diabetes [32]. The dynamics of postmenopausal gonadotropin secretion are not fully understood, and data of T2D women are scarce and contradictory [33,34]. FSH has been shown to have direct effects on bone remodeling in rodents, independent of estrogen levels [35]. Therefore, additional studies are needed to assess whether changes in the hypothalamic-pituitary-gonadal axis account for our observation of reduced serum FSH levels in diabetic women, and to investigate what effects might be expected on bone metabolism.

Mean baseline levels of bone remodeling markers OC and CTX were lower in participants with T2D than in controls, which would indicate that diabetic women had reduced turnover. The observed relationship between OC and T2D might be at least partially explained by BMI, which was higher in group T2D. Indeed, Viljakainen and colleagues have recently found that the response of various bone remodeling markers, including OC and CTX, to the oral glucose tolerance test was lower among severely obese individuals in comparison to controls [36]. Until the relationship between T2D and bone turnover markers is fully elucidated, therapeutic decision-making based on assessment of these markers (for example, in the treatment of osteoporosis) should be taken with care in diabetic individuals.

Serum CTX dynamics in response to the mixed meal was different in T2D participants in relation to controls, suggesting that nutrient-dependent suppression of bone resorption is attenuated in T2D. Indeed, Chailurkit and colleagues have reported similar findings using the oral glucose tolerance test [29]. Plasma OC levels decreased similarly throughout the mixed meal tolerance test in both groups, however they were persistently lower in T2D subjects. Importantly, body mass index did not seem to influence the comparison of CTX and OC response between groups.

Confirming the role of nutrient intake as a stimulating factor for GLP-2 production [12,14,37], GLP-2 response to the mixed meal was similar in both groups. Therefore, based on the results of this study, we cannot ascertain that GLP-2 is responsible for the abnormal bone remodeling in T2D. Nevertheless, we found a positive correlation between GLP-2 and femoral neck and total hip BMD, corroborating a potential influence of this intestinal hormone on bone metabolism. Further clinical studies are necessary to clarify the role of GLP-2 in bone metabolism in healthy and T2D individuals.

## Conclusion

We conclude that T2D postmenopausal women have altered bone remodeling at basal conditions and in response to nutrient intake. Further studies focusing on the influence of gastrointestinal hormones on bone metabolism are needed to advance our understanding of bone fragility in individuals with diabetes.

## Abbreviations

ADA: American Diabetes Association
ANOVA: Analysis of variance
BMD: Bone mineral density
BMI: Body mass index
CO: Control
CTX: C-terminal telopeptide of type I collagen
DXA: Dual-energy x-ray absorptiometry
FSH: Follicle-stimulating hormone
GLP-2: Glucagon-like peptide 2
HbA1c: Glycated hemoglobin
LH: Luteinizing hormone
OC: Osteocalcin
PTH: Parathyroid hormone
T2D: Type 2 diabetes mellitus
